# Use of Zebrafish Larvae as a Multi-Endpoint Platform to Characterize the Toxicity Profile of Silica Nanoparticles

**DOI:** 10.1038/srep37145

**Published:** 2016-11-22

**Authors:** Duc-Hung Pham, Bert De Roo, Xuan-Bac Nguyen, Mattias Vervaele, Angela Kecskés, Annelii Ny, Daniëlle Copmans, Hanne Vriens, Jean-Pierre Locquet, Peter Hoet, Peter A. M. de Witte

**Affiliations:** 1 Laboratory for Molecular Biodiscovery, KU Leuven, Campus Gasthuisberg, Herestraat 49, O&N II, 3000 Leuven, Belgium; 2 Solid State Physics and Magnetism Section, KU Leuven, Department of Physics and Astronomy, Celestijnenlaan 200D, 3001 Heverlee, Belgium; 3 Center for Environment and Health Leuven, KU Leuven, Campus Gasthuisberg, Herestraat 49, O&N I, 3000 Leuven, Belgium

## Abstract

Nanomaterials are being extensively produced and applied in society. Human and environmental exposures are, therefore, inevitable and so increased attention is being given to nanotoxicity. While silica nanoparticles (NP) are one of the top five nanomaterials found in consumer and biomedical products, their toxicity profile is poorly characterized. In this study, we investigated the toxicity of silica nanoparticles with diameters 20, 50 and 80 nm using an *in vivo* zebrafish platform that analyzes multiple endpoints related to developmental, cardio-, hepato-, and neurotoxicity. Results show that except for an acceleration in hatching time and alterations in the behavior of zebrafish embryos/larvae, silica NPs did not elicit any developmental defects, nor any cardio- and hepatotoxicity. The behavioral alterations were consistent for both embryonic photomotor and larval locomotor response and were dependent on the concentration and the size of silica NPs. As embryos and larvae exhibited a normal touch response and early hatching did not affect larval locomotor response, the behavior changes observed are most likely the consequence of modified neuroactivity. Overall, our results suggest that silica NPs do not cause any developmental, cardio- or hepatotoxicity, but they pose a potential risk for the neurobehavioral system.

In recent years, nanotechnology has rapidly gained interest for innovative applications in several areas. In the healthcare sector, nanotechnology-based products are already extensively used. Since 2006, around 250 nanotechnology-based drugs, delivery systems and imaging devices were in preclinical, clinical and commercial development^1^.

Among nanomaterials, nanoform silica is one of the top 5 nanoparticles (NPs) and is used mostly in consumer products such as food and drugs^2^. Recently, individual consumer intake of silica from food was estimated at 9.4 mg/kg bw/day, of which around 20% (1.8 mg/kg bw/day) was estimated to be in the nano-size range^3^. Remarkably, as much as 43% of silica of synthetic origin present in food products is in the nanometer size range^3^,^4^. In addition, numerous forms of silica nanoparticles, such as amorphous or mesoporous silica, are under development for biomedical applications including medical diagnostics, drug delivery, gene therapy, biomolecules detection, photodynamic therapy and bioimaging^5^,^6^.

The increased production and use of NPs may pose a significant and unexpected risk to humans and the environment since they often have different physicochemical, optical and electrical characteristics as compared to their bulk counterparts^7^. NPs may infiltrate many body compartments and interact directly with macromolecules in the body such as DNAs, RNAs or proteins in different ways. Many studies suggest that the size and surface or physical characteristics of NPs play a more important role in the toxicity of NPs than in case of their equivalent macroscopic materials^8^.

Zebrafish are emerging as a small animal model for genetic, developmental, pharmacological and toxicological research. The zebrafish genome has been fully sequenced and shows close homology to human genome^9^. Zebrafish larvae are transparent, and their early organogenesis can be observed using simple light microscopy. One adult pair of zebrafish can produce up to 200–300 embryos per mating^10^. In addition, zebrafish larvae fit in 96 or 386-wells plates, enabling high throughput assays. Several studies have validated the use of zebrafish larvae as a model for cardio-, hepato- and neurotoxicity research^11^,^12^. Currently, zebrafish larvae or embryos are being used as *in vivo* platforms to study the toxic effects of nanoparticles^13^,^14^. Table 1 provides a literature overview of studies on the toxicity of nanomaterials as a function of their physicochemical characteristics using zebrafish models. However, the toxicity endpoints used in most studies are only based on external phenotypic changes^13^, whereas toxic effects on internal organs were not assessed. Moreover, in *in vivo* systems, tissues and cells are constantly interacting with each other. For instance, in zebrafish there is evidence that the cardiovascular system affects the normal development of the liver while neurobehavior is not determined only by neurons but also by the muscular system^15^. Therefore, the zebrafish model may allow to observe harmful effects of NPs in specific organs, secondary to the toxicity elicited in other organs.

In our study, we used fertilized eggs and larvae of zebrafish as a versatile platform to test the effect of silica NPs on time of hatching and survival and investigate whether they induce malformation, cardio-, hepato- and neurotoxicity. To gain better insight into the effects of silica NPs with respect to size, if any, we chose three diameters (i.e. 20 nm, 50 nm, 80 nm) for toxicity testing. Our approach can provide the basis for a high throughput platform to study the toxicity of not only silica but also many other NPs. These data will in turn be useful in managing risk and reducing hazardous effects of NPs, as well as supporting “safe-by-design” strategies of industrial nanotechnology-based companies.

## Results

### Silica NPs characterization

Silica NPs dispersed in Milli-Q water was obtained from Nanocomposix (SanDiego, USA). Upon arrival, we characterized the material using dynamic light scattering (DLS), small-angle X-ray scattering (SAXS), transmission electron microscopy (TEM) (Supplementary Figure S1) and inductively coupled plasma mass spectrometry (ICP-MS). The results are summarized in Table S1 (Supplementary). The results show that the size of stock silica NPs are consistent with the ones declared by the manufacturer. Hereafter, 20, 50 and 80 nm silica NPs are denoted as S-20, S-50 and S-80.

Since silica NPs were diluted in zebrafish medium (Danieau) before being tested on their toxicity, the size of silica NPs in these conditions was also monitored. The DLS data summarized in Table 2 show a slight decrease in size after storage of the nanoparticles at room temperature. Moreover, the stability of a concentration (i.e. 200 mg/l) of silica NPs in Danieau’s was investigated as a function of time. The measurements were done by comparing the intensity of the extrapolated scattering intensity at a 0 scattering angle. The data obtained with SAXS were consistent and did not show a notable decrease in concentration over 5 days (Table 2). Therefore, we concluded that the silica NPs were stable in Danieau’s medium over the course of our experiments.

### Quantification of silica NP associated with chorion and embryo

**Figure 1:**
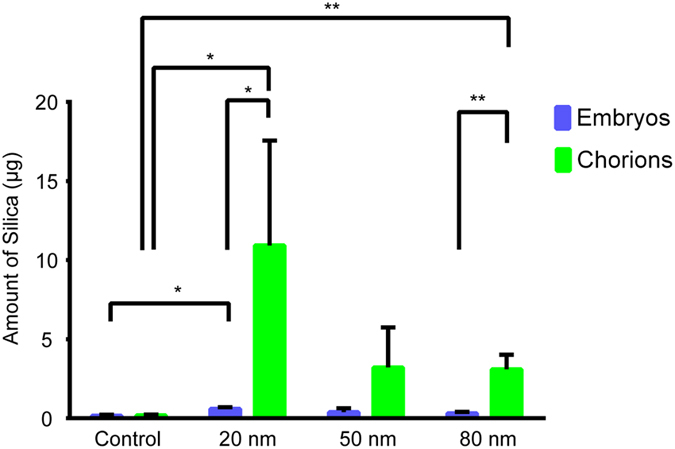
Quantification of silica NP associated with chorion and embryo. Amount of silica (μg) measured by ICP-MS associated with chorions and embryos after exposing fertilized eggs to 20 nm, 50 nm and 80 nm silica NPs at a concentration of 200 mg/l from 4hpf to 24hpf. A group of 50 fertilized eggs was used per condition. Data represent the means and standard deviations for at least three biological replicates. (*p < 0.05, **p < 0.01, student t-test).

Fertilized eggs were exposed to S-20, S-50 and S-80 at a concentration of 200 mg/l from 4hpf to 24hpf. At 24hpf, eggs were dechorionated and the amount of silica associated with the separated chorions and embryos examined. As shown in Fig. 1, the content of silica in control embryos and chorions was minimal whereas that of the silica NPs-treated groups was much higher. In case of S-20 and S-80 the amount of silica associated with the chorion (i.e. 10.93 ± 6.63 μg and 3.10 ± 0.92 μg, respectively) (data presented as mean ± SD) was significantly higher as compared to the control value (0.19 ± 0.05 μg). In case of S-50, the amount of silica adhering to the chorion (3.22 ± 2.52 μg) was not significantly different from that of the control. In general, the amount of silica associated with the embryos was much lower (0.60 ± 0.10 μg, 0.39 ± 0.24 μg and 0.31 ± 0.10 μg for S-20, S-50 and S-80, respectively). Only in case of S-20 a statistically significant difference was found with the control value (i.e. 0.16 ± 0.07 μg).

### Developmental toxicity

#### Survival and time of hatching

**Figure 2:**
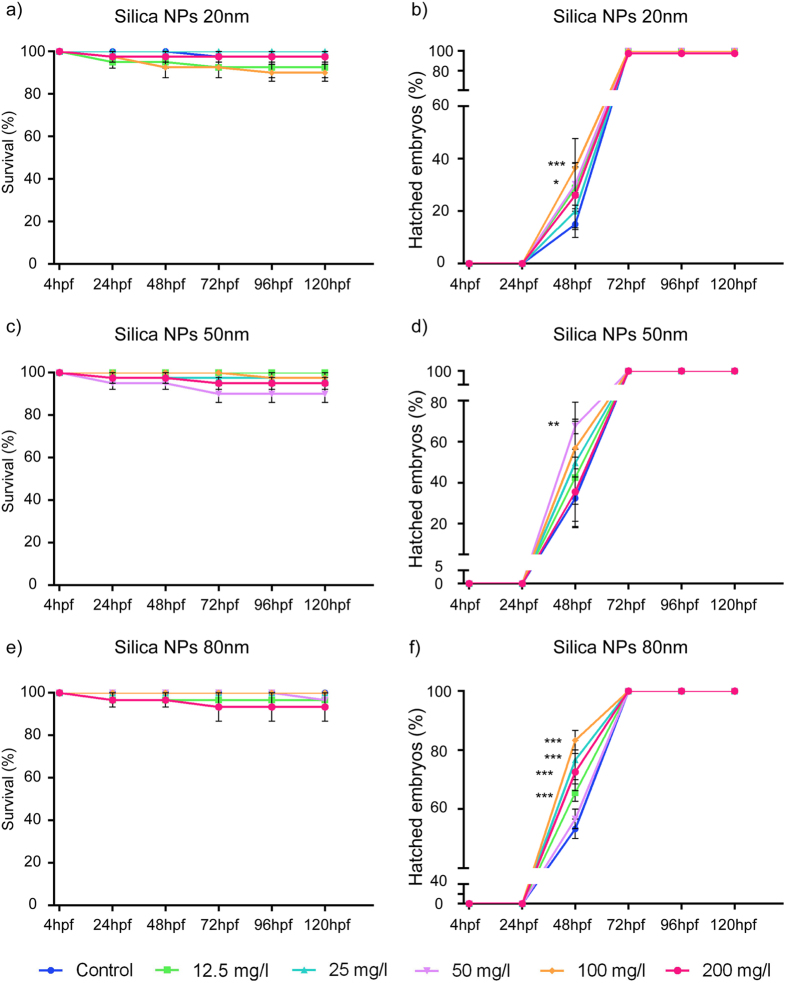
Developmental toxicity of silica NPs. Effect of 20 nm (**a**,**b**), 50 nm (**c**,**d**) and 80 nm (**e**,**f**) silica NPs on survival (**a**,**c**,**e**) and hatching time (**b**,**d**,**f**). Fertilized eggs were exposed to the NPs from 4hpf–120hpf using concentrations ranging from 12.5 mg/l to 200 mg/l. 10 eggs were used individually per condition, and the result expressed as percentage of the total amount of embryonic/larval fish that survived (**a**) or total amount of embryos that hatched (**b**) as a function of time. The data represent the means and standard errors of the means for at least three independent biological replicates. (*p < 0.05, **p < 0.01, ***p < 0.001, two-way ANOVA, Bonferroni correction).

Survival and time of hatching of zebrafish embryos and larvae were observed and followed throughout the whole exposure period (4hpf–120hpf) (Supplementary Figure S2a). As shown in Fig. 2, the survival curves recorded for S-20, S-50 and S-80 were similar and not significantly different from the controls.

The silica NPs however altered the hatching kinetics of the embryos (Fig. 2b,d and f). In control conditions hatching started between 48hpf to 78hpf. Specifically, S-80 accelerated the time of hatching significantly at almost all concentrations (12.5, 25, 100 and 200 mg/l) (Fig. 2f). S-50 and S-20 also showed similar accelerated hatching kinetics with the effects only statistically significant at concentrations of 50 and 100 mg/l (Fig. 2b,d).

#### Morphology

The embryos and larvae were also scored for morphological defects throughout the whole exposure period using a semi-quantitative scale (Supplementary Figure S3a). In a small percentage of the animals abnormalities were seen that included pericardiac edema, yolk sac edema, delayed growth, bent spine and opaque tissue. However, at 120hpf, the percentage of normal larvae in silica NPs-treated groups was not statistically different from that of the control conditions (Supplementary Figure S3b). Similar results were obtained in the 24 hpf–96hpf period (data not shown).

#### Heart and liver toxicity

**Figure 3:**
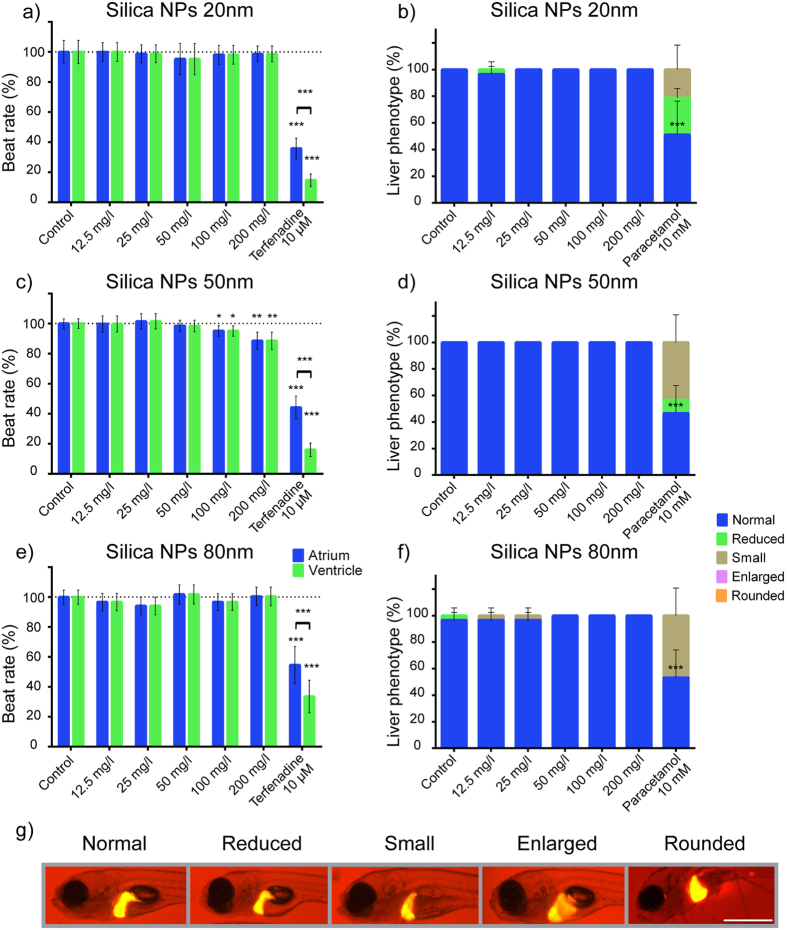
Heart and liver toxicity of silica NPs. Effect on atrium and ventricle beat rate (**a**,**c**,**e**) and liver phenotype (**b**,**d**,**f**) of 20 nm (**a**,**b**), 50 nm (**c**,**d**) and 80 nm (**e**,**f**) silica NPs. Transgenic *Tg*(*fabp10a:Dsred*) 4dpf larvae were treated with silica NPs using concentrations ranging from 12.5 mg/l to 200 mg/l and at 5dpf and 7dpf examined for beat rate and liver phenotypes (g, scale bar = 0.5 mm), respectively. Terfenadine (10 μM) and paracetamol (10 mM) were used as positive controls. In case of beat rate measurements, 10 larvae were used individually per condition and results normalized against that of control larvae. The data represent the means and standard deviation for the pooled data (10 larvae, three independent biological replicates, n = 30). In case of liver phenotyping, 10 eggs were used individually per condition, and the result expressed as percentage of the total amount of larvae present that had a normal or abnormal liver (i.e. small, shrunk, swollen, spherical shape). The data represent the means and standard deviation for three independent biological replicates. (*p < 0.05, **p < 0.01, ***p < 0.001, two-way ANOVA, Bonferroni correction).

For testing heart and liver toxicity, 4dpf larvae of the transgenic line *Tg*(*fabp10a:Dsred*)^15^ were treated with silica NPs (Supplementary Figure S2b). After 24 h exposure to silica NPs 5dpf larvae were examined for heart rate abnormality. As shown in Fig. 3, we did not observe any significant change in the heart rate of larvae treated with S-20 and S-80, but a significant trend towards a decreased rate was seen with S-50 at the highest concentrations (Fig. 3c). Conversely, in terfenadine-treated larvae (positive controls), the heart rates of atrium and ventricle were reduced significantly in comparison with those of the untreated larvae and in addition arrhythmia was observed.

At 7dpf, larvae were screened for their liver phenotypes. The number of larvae with normal liver phenotypes in silica NPs-treated groups was not significantly different from the control larvae group (Fig. 3b,d and f). On average, in S-20 and S-50 experiments, 100% (+0.0, SD) of the control larvae had a normal liver whereas in the S-80 experiment, 96.7% (+5.8) of the control larvae showed a normal liver morphology. In the group treated with paracetamol, a well-established compound causing liver toxicity, only about half the larvae had normal livers (51.1% (+25.3), 46.7% (+20.8), and 53.3% (+20.8), in the S-20, S-50 and S-80 experiments, respectively) while the rest showed livers of reduced and small size.

### Neurotoxicity

#### Photomotor response (PMR)

**Figure 4:**
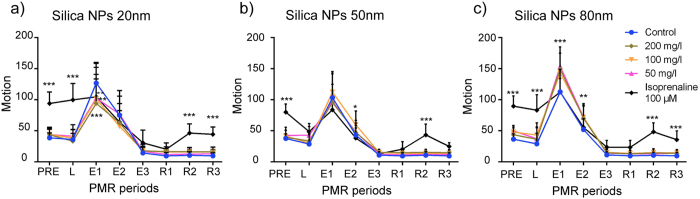
Neurotoxicity of silica NPs as measured by the photomotor response (PMR) assay. PMR was conducted on 30hpf embryos exposed between 4hpf–30hpf to silica NPs using concentrations ranging from 50 mg/l to 200 mg/l. The locomotor behaviors of embryonic zebrafish within the chorion was divided into the pre-stimulus (PRE), latency (L), excitation (E1, E2, E3) and refractory (R1, R2, R3) phases. 100 μM isoprenaline was used as a positive control. Each well of a 96-well plate contained five fish and three to six wells were used per condition. Raw data of total movement per well were used (expressed in motion units) and the data obtained from the different wells per condition averaged. Data represent the means and standard deviations for three independent biological replicates. (*p < 0.05, **p < 0.01, ***p < 0.001, two-way ANOVA, Bonferroni correction).

For early neurotoxicity testing, fertilized eggs were exposed to silica from 4hpf–30hpf and then subjected to the PMR assay^16^ (Supplementary Figure S2a). As shown in Fig. 4 (and Supplementary Figure S4), silica NPs changed PMR in various ways but mainly at the E1 and E2 excitation phases. S-20 significantly suppressed PMR in a concentration-dependent manner at the E1 phase (Fig. 4a). Similarly, S-50 also induced a modified PMR, but the effect was only significant at the E2 phase at 100 mg/l (Fig. 4b), whereas S-80 increased PMR significantly during the E1 and E2 phases to a comparable level at three concentrations (50, 100 and 200 mg/l) (Fig. 4c).

All embryos showed normal touch response (data not shown).

#### Locomotor response (LMR)

**Figure 5:**
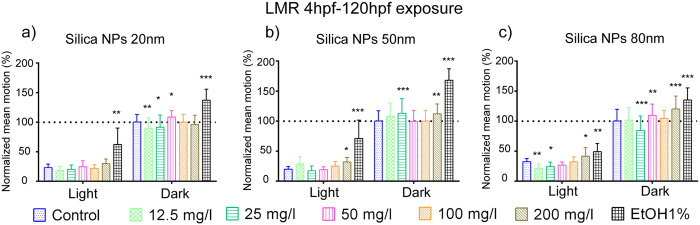
Neurotoxicity of silica NPs (long exposure) as measured by the locomotor response (LMR) assay. LMR during light-dark cycles was conducted on 5dpf larvae exposed between 4hpf–120hpf to silica NPs using concentrations ranging from 12.5 mg/l to 200 mg/l. The data from the first 10 min light and dark periods were omitted (acclimation). 1% ethanol was used as positive control. Experiments were repeated independently three times. The raw data of the total movement per minute time interval of 10 larvae per condition (i.e. different silica concentrations, controls) were averaged and the averages per condition for the combined light (i.e. totally 15 min) and dark conditions (i.e. totally 20 min) of the three experiments pooled. The mean of the control values obtained in dark conditions was set to 100% and the results of the other conditions normalized. Data represent the means and standard deviations (*p < 0.05, **p < 0.01, ***p < 0.001, two-way ANOVA, Bonferroni correction).

Following the silica NP exposure period (4hpf–120hpf), neuroactivity of zebrafish larvae was also analyzed by the LMR assay to test any locomotor defects resulting from modified neuroactivity. In the light-dark alternating tracking test, larvae move more in the dark and less in the light period^17^. Control larvae responded accordingly, but silica NPs-treated larvae showed in general a modified LMR (Fig. 5, Supplementary Figure S5). In case of S-20, the LMR was not modified in the light period. In the dark period however, we observed that at the lowest concentrations (12.5 and 25 mg/l), LMR was significantly decreased, whereas the response was significantly increased at 50 mg/l (Fig. 5a and Supplementary Figure S5a). Conversely, S-50 increased the LMR significantly in both the light and dark periods, but only at 200 mg/l (light) and at 25 and 200 mg/l (dark) (Fig. 5b and Supplementary Figure S5b). Also S-80 had a significant influence on LMR in both light (at 12.5, 50 and 200 mg/l) and dark conditions (at 25, 50 and 200 mg/l) (Fig. 5c and Supplementary Figure S5c).

As shown in Fig. 2, silica NPs also accelerated the hatching time of the zebrafish embryos. To exclude the possibility that the changes in LMR of 5dpf larvae were affected by this acceleration, we conducted the LMR assay using 48hpf embryos that were already hatched and 48hpf embryos that were dechorionated before. However, 120hpf larvae of both groups did not change their LMR in the light and dark period (Supplementary Figure S6a,b).

**Figure 6:**
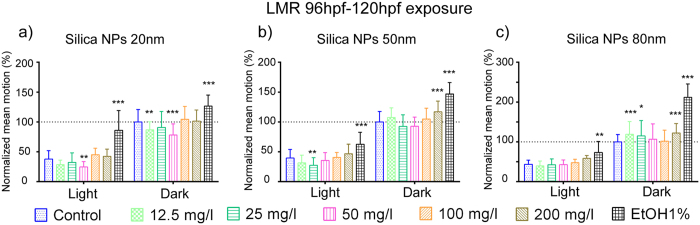
Neurotoxicity of silica NPs (short exposure) as measured by the locomotor response (LMR) assay. LMR during light-dark cycles was conducted on 5dpf larvae exposed between 96hpf–120hpf to silica NPs using concentrations ranging from 12.5 mg/l to 200 mg/l. The data from the first 10 min light and dark periods were omitted (acclimation). 1% ethanol was used as positive control. Experiments were repeated independently three times. The raw data of the total movement per minute time interval of 10 larvae per condition (i.e. different silica concentrations, controls) were averaged and the averages per condition for the combined light (i.e. totally 15 min) and dark conditions (i.e. totally 20 min) of the three experiments pooled. The mean of the control values obtained in dark conditions was set to 100% and the results of the other conditions normalized. Data represent the means and standard deviations (*p < 0.05, **p < 0.01, ***p < 0.001, two-way ANOVA, Bonferroni correction).

We then performed another LMR experiment, in which 96hpf larvae were treated with silica NPs for 24 h, after which they were subjected to LMR testing (Fig. 6 and Supplementary Figure S7). Also in this case, the exposure to silica NPs resulted in a slightly modified LMR, with a similar variability in the responses (Fig. 6 and Supplementary Figure S7). Specifically, S-20 decreased LMR in the light and dark periods, and the effect was significant at 12.5 and 50 mg/l (dark) and 50 mg/l (light) (Fig. 6a). In case of S-50 nm silica NPs also a significant LMR decrease in light conditions was observed (at 25 mg/l), whereas an increased response was seen that was significant at 200 mg/l in the dark period (Fig. 6b). S-80 altered LMR in the light and dark periods to a similar extent. In the light period, the results showed a trend to an increased LMR, even though the effect was not significant. In the dark period, S-80 at 12.5, 25, and 200 mg/l increased LMR significantly (Fig. 6c).

All embryos showed normal touch response (data not shown).

## Discussion

As nanomaterials are produced worldwide in increasingly large quantities, environmental and human exposures are inevitable. Unfortunately the potential hazards of these materials that do not only feature different chemical composition, but also come in different sizes and with specific physicochemical and electrical characteristics^7^, often remain completely unidentified. Hence there is an unmet need for fast, sensitive and cheap *in vivo* models that act as safety testing platforms to reliably predict the complexity of responses to nanoparticles in humans.

Exposure to both natural and engineered NPs can arise fom a variety of sources^18^,^19^. A recent assessment on the global life cycle of engineered NPs demonstrated that a significant fraction is present in soil (8–28%), water (0.4–7%) and the atmosphere (0.2–1.5%); and silica NPs are known to be among the most abundant components in the global life cycle of engineered NPs^19^,^20^. Synthetic silicon-based compounds are often added to food, but silicon also occurs as a natural component in foodstuffs in the form of sodium, calcium and magnesium silicates or as hydrated silica SiO_2_-nH_2_O^21^. The latter may form small (*i.e.* 1 nm to 5 nm) particles that can be found in natural and drinking waters^4^. Hence, as the concentrations of silica present in nature are complex and variable, we used a concentration range to be tested in zebrafish larvae (i.e. from 12.5 mg/l to 200 mg/l) which is in line with the OCECD guideline for fish acute toxicity testing^22^.

In this study we have investigated the toxicity of silica nanoparticles of three different sizes (20 nm, 50 nm, 80 nm) using zebrafish larvae as a model that assesses multiple toxicological endpoints. We obtained these silica NPs from a commercial source (Nanocomposix) and characterized them by DLS, SAXS, TEM, ICP-MS and zeta potential measurements. Our results confirm the data supplied by the manufacturer concerning the characteristics of the silica NPs. Furthermore, we monitored the stability and concentration of the nanoparticles in Danieau’s solution during 5 days which corresponds to the experimental time period.

The DLS data showed a slight decrease in size after storage of the nanoparticles at room temperature. This can be explained by the higher ionic strength of Danieau’s medium compared with Milli-Q water that decreases the Debye length resulting in a reduction of the hydrodynamic radius of the nanoparticle. Notably, a decrease in the Debye length can also result from a decreased electrostatic repulsion of the nanoparticles leading to aggregation. This possibility however was excluded by the fact that no change in polydispersity index (PDI) was observed. Overall, the results show that the nanoparticles do not aggregate as the PDI values did not change and as the hydrodynamical radius only decreased slightly over time. Furthermore, the DLS and SAXS data show that the silica NPs tested were uniform in size over time and that stable concentrations were obtained.

During the developmental cycle of zebrafish, earlier developing embryos are more sensitive to xenobiotic substances compared to juveniles or adults^23^. Therefore, we chose 4hpf–120hpf as a suitable time window for exposure of zebrafish larvae to silica NPs to dissect any possible developmental toxicity.

In this study, we did not observe any significant mortality or malformation of zebrafish embryos/larvae induced by silica NPs. However, in general silica NPs accelerated the time of hatching of the embryos. This phenomenon was most apparent for 80 nm silica NPs (S-80) while 50 nm and 20 nm silica NPs (S-50, S-20) also promoted hatching but to a lesser degree and without a dose-dependent effect. As the pores of the chorion have sizes between 0.3–0.7 μm, many small NPs can adsorb onto its external surface^24^,^25^, and it has been suggested that the blockage of these pores by NPs hinder respiration and the excretion of metabolites. The increased respiration rate then can facilitate the release of enzymes related to hatching and rupture of the chorion^26^. This may also explain the accelerated hatching in our study as we found a high amount of silica associated with chorions at 24hpf (Fig. 1).

Our results concur with another study of 60 nm and 200 nm fluorescent silica nanoparticles (FSNP) in zebrafish larvae^25^. This study showed that neither 60 nm nor 200 nm FSNP adversely effected the embryonic development, while also reporting a promotional effect on the time of hatching of zebrafish embryos. Of interest, the latter has also been observed when zebrafish embryos are treated with titanium dioxide NPs^27^.

In contrast, using home-made silica nanoparticles with an average diameter of 62 nm Duan *et al*.^28^ found an inhibitory effect on the hatching rate. It is possible that these conflicting results are due to the different surface and physicochemical characteristics of the silica NPs used in both studies. On the other hand, as they are highly influenced by the conditions used, hatching rates are notoriously variable (as also seen in this study) and consequently hatching rate data should be interpreted with caution. In our experiments silica NPs were refreshed for 40% every 24 h, and although we took great care not to disturb the embryos during the removal and replacement of the liquid (no mixing performed), it is possible that these manipulations have influenced the overall outcome of the experimental work.

While silica NPs in our experimental conditions did not induce any overt sign of malformation, we assessed if they could cause any damage to internal organs. Among these, the heart is the first to form and start functioning. Notably, an association exists between short-term elevations in PM2.5 (particulate matter <2.5 μm in aerodynamic diameter) and increased heart disease-related mortality^29^. In this study, S-20 and S-80 did not cause any significant heart rate abnormality, while S-50 induced a concentration dependent decrease in the heart rate. However, even at the highest concentration, the maximum reduction in heart rate of larvae exposed to S-50 was limited to 12%. As a consequence we did not observe any pericardiac edema nor yolk sac edema, as these functional symptoms of bradycardia are expected only when the resting heart rate is reduced by at least 20%^12^.

Our data therefore show that some toxicological effects of NPs can vary with the particle size. For instance, also in the PMR assay (see further) the S-80, S-50 and S-20 show a clear differential activity. Although these observations remain unexplained, our results indicate that it is essential to comparatively study different sizes of nanoparticles as their specific physicochemical characteristics might influence dramatically their biological effects.

The present outcome on NPs and cardiotoxicity is also in line with the results of another study using silica nanoparticles that induced significant bradycardia in zebrafish embryos at concentrations similar to the ones that exerted an effect in our study (i.e. 100 mg/l, 200 mg/l)^30^. Unfortunately, no other particle sizes were examined but it is interesting to note that the nanoparticles used had an average diameter of 62 nm that is close to the diameter (50 nm) of the NPs that showed activity in our study.

Since the liver is key to the metabolism of xenobiotics, and drug-induced liver injury is the main cause of attrition in post-marketing surveillance^31^, we also tested silica NPs for their effect on the zebrafish livers. Currently, only one study using a zebrafish transgenic line *Tg*(*hsp70:GFP*) reported on liver toxicity induced by gold NPs as indicated by the expression of heat shock protein 70^32^. Importantly, in zebrafish larvae the growth of liver tissue depends critically on normal endothelial and cardiovascular systems^15^, and it can be expected that observable effects on these structures would indicate liver toxicity. Our results clearly show that silica NPs did not decrease the number of larvae with normal liver phenotypes, and hence the outcome is somewhat contradictory to previous studies using mice and rats showing that silica NPs induce spleen, lung and liver damage. However, in the rodent studies liver toxicity was only observed after silica NPs were administrated intravenously^33^, maternally transferred^34^ or when the animals were orally given very high chronic doses^4^, which may not be representative of real life exposure.

Beside heart and the liver, brain is also a possible target organ of NPs. It has been demonstrated that in fish and mammals, NPs may enter the brain through the nerve endings of the olfactory bulbs^35^,^36^. Currently, very little information is available regarding the neurobehavior effects of NPs in fish. To our knowledge, this is the first study that uses a zebrafish PMR assay to describe NP-induced toxicity. While PMR is used in drug-discovery screenings for neuroactive compounds^37^, PMR-based toxicity investigations are limited. In one recent publication, PMR was used as one of the useful endpoints for hazard assessment of chemicals in US ToxCast program^38^. That study also demonstrated that embryonal zebrafish is a useful model for flagging neurotoxicants.

Our present work shows that silica NPs modified the photomotor activity of early non-hatched embryos as measured by the PMR assay (Fig. 4). While S-80 and S-50 induced a clear and mild hyperactivity, respectively, S-20 caused hypoactivity. As PMR behavior is primarily triggered by a discrete set of neurons within the embryo hindbrain^39^, it is possible that these effects are due to a direct exposure of brain tissue to the NPs. Notably, in this study we were able to quantify small amounts of silica associated with the embryos after incubation of the fertilized eggs. Possibly this silica was taken up by the embryos by an internalisation process, but although steps were taken to minimize any contamination, we presently cannot rule out the possibility that some transfer took place during the dechorionation manipulations from the chorions highly loaded with silica to the embryonal tissue. Hence, whether silica NPs modified the PMR outcome by a direct effect on brain tissue or via indirect effects (e.g. by association with the chorion) is presently unknown.

Moreover, silica NPs also altered locomotor activity of developing embryos/larvae after long-term (4hpf–120hpf) or short term (96hpf–120hpf) larval exposure (Figs 5 and 6), although the individual effects of the different concentrations of the different NP sizes were variable. Overall, the results are therefore in line with the results of the PMR assay. Hence our results are consistent with previous studies showing that silver and titanium dioxide NPs exhibited an obvious dose and size-dependent effect on the larval swimming behavior^40^,^41^.

Our findings also show that silica NPs accelerate the time of hatching of zebrafish embryos while changing the PMR and LMR of zebrafish embryos and larvae. Hence, the relationship between early hatching and the alteration of LMR was investigated. We were able to demonstrate that early hatching and dechorionation are not motoractivity-modifying factors. Locomotor behavior of zebrafish is influenced by many factors, including neurotoxicity, neuromuscular or physiological injury. Gill injury and subsequent hypoxia have been suggested as the major reason for the decreased swimming speed in rainbow trout exposed to titanium dioxide NPs^42^. In all our experiments, only zebrafish scored as “normal” in the semi-quantitative scale were included in the PMR/LMR assay. Besides, the embryos/larvae also showed a normal touch response indicating a normal neuromuscular activity. Therefore, the altered PMR/LMR of zebrafish in this study is most likely the result of some neurobehavioral toxicity induced by silica NPs in developing fish.

It was reported that 15 nm silica NPs induce changes in the morphology of neuroblastoma cells and increases deposition of intracellular β-amyloid^43^. Moreover, silica NPs can induce brain accumulation of particles, increase oxidative stress and pro-inflammatory cytokines after intranasal instillation^44^ or result in behavioral impairment in rats^45^. In zebrafish adults, 15 nm silica NPs significantly decreased dopaminergic neuron level and behavioral activities, neither of which was observed for 50 nm NPs^46^. Besides, it is worth noting that while 15 nm silica NPs were found to significantly reduce dopamine level in the striatum, they also increased the glutamate content in the hippocampus^44^. Interestingly, our study showed that 20 nm silica NPs caused hypoactivity while 50 nm and 80 nm silica NPs induced hyperactivity. Whether a correlation exists between the size of silica NPs and the balance of neurotransmitters (dopamine *vs* glutamate) that underlies the altered locomotor activity in exposed zebrafish, possibly as the result of a differential accumulation in substructures of the larval brain, is an interesting hypothesis that warrants further investigation.

Taken together, toxicological effects observed in this study as assessed by a zebrafish platform that analyzed multiple endpoints were mild or even absent. Likely, in some cases the combination of the relatively small changes induced by the particles (as observed in the hatching rate, LMR and PMR assays) with a degree of variability in the biological response, caused some inconsistencies in the concentration-response relationships. Indeed, we have seen that depending on small changes in the standard deviations some means reached statistical significance whereas others did not. For instance, 50 mg/l was the only concentration that did not show an effect in S-80 accelerated hatching. In addition, also the individual results of the LMR assay showed some variability, although overall a clear effect could be observed. The present study therefore also shows that in case of limited toxicity generated by particles of compounds, it is essential to examine a large variety of conditions in order to increase the predictive power of the model and to find statistically significant trends.

In conclusion, by using zebrafish larvae as a model, we have characterized the toxicity profile of silica NPs with different sizes. With this model, not only the overt sign of toxicity (e.g. developmental malformation, mortality) but also the internal organ damage (e.g. cardio-, hepato- and neurotoxicity) can be investigated. Our model offers a medium/high through-put but thorough platform for characterizing toxicity profiles of nanomaterials. Future experiments will help to understand better the mechanism underlying the accelerated hatching and neurobehavior effects as observed in this study for silica NPs.

## Methods

### Ethics

All zebrafish experiments carried out were approved by the Ethics Committee of the University of Leuven (approval number 061/2004) and by the Belgian Federal Department of Public Health, Food Safety & Environment (approval number LA1210199). All procedures were carried out according to the Declaration of Helsinki and conducted according to the guidelines of the European Community Council directive 86/609/EEC.

### Silica NPs acquisition

Nanoparticles were purchased from Nanocomposix (San Diego, USA) in three different sizes: 20 nm, 50 nm and 80 nm which were dispersed in Milli-Q water. Detailed characterization of those silica NPs were given by the manufacturer (Supplementary Table S1). The nanoparticles were used as received without any further purification.

### Silica NPs characterization

The silica NPs were characterized by DLS, SAXS, TEM, ICP-MS and zeta potential measurements.

A VASCO particle size analyzer DL135 (Cordouan Technologies) was used to determine the hydrodynamic radius of the nanoparticles. The samples were diluted as needed with deionized water to obtain a suitable scattering intensity (2500 kcps). DLS measurements were achieved with Cordouan’s NanoQ proprietary software. The DLS technique is based on recording the scattered intensity which fluctuates randomly due to the Brownian motion of NPs in suspension. The hydrodynamic radius of NPs is deduced from the intensity fluctuation auto-correlation and calculated with the Padé-Laplace method.

For SAXS measurements a Nanostar (Bruker AXS) was used, which features the novel MetalJet X-ray source (Excillum). This X-ray source functions with liquid metal i.e. an alloy of gallium and indium, as target material^47^ and SCATEX pinholes (Incoatec). The radiation consists of Gallium Kα X-rays which have a wavelength of 1.34 Å. A more detailed description of this setup can be found in ref. 48. After background correction, the SAXS data was fitted to extract the size distribution. The fit function is calculated according to equation 1:


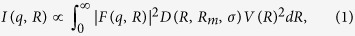


Here, q = 4π/λ sin θ, which is the modulus of the scattering vector as a function of the wavelength λ and the scattering angle 2θ. The latter is the angle between the wavevector of the incident and scattered beam. D (r, rm, sigma) is a Gaussian size distribution function.We fitted the data with the form factor of a sphere^49^ according to equation 2:


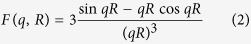


TEM measurements were performed with a transmission electron microscopy (CM 200FEG Philips) operating at 200 kV. The TEM grids were prepared by placing 8–10 μl of particle solution on a carbon-coated copper grid and dried under ambient conditions. At least 100 particles were analyzed for determination of the mean radius and standard deviation. Those were automatically analyzed with ImageJ and the psa macro.

Zeta potential measurements were carried out with a Zeta Potential WALLIS (Cordouan Technologies) equipped with a 20 mW diode at 635 nm coupled to an automated optical attenuation system. Standard glass cuvettes (Helma) were used. The samples were sonicated prior to the measurement for 5 minutes. 10 measurements were done and the average of these results was used.

For ICP-MS measurements, 1 ml of nitric acid (HNO_3_) 60% ultrapur (Merck, Darmstadt, Germany) was added to 1 ml of Danieau’s medium (1.5 mM HEPES, pH 7.6, 17.4 mM NaCl, 0.21 mM KCl, 0.12 mM MgSO_4_, and 0.18 mM Ca(NO_3_)_2_) and heated at 180 °C for 5 hours. Finally, solutions were 10 times diluted in distilled water and metal concentration (silicium) was measured by ICP-MS (Agilent 7700x ICP-MS).

### Zebrafish maintenance and silica NPs exposure protocols

Adult fish (0.5–1 year old, mixed sexes) were maintained in a UV-sterilized rack recirculating system equipped with a mechanical and biological filtration unit and kept under a 14/10 hour light/dark cycle at the temperature of 27–28 °C and pH of 6.8–7.5. Water quality was monitored daily for pH, temperature and conductivity, and weekly for ammonia and nitrite levels (SL1000 Portable Parallel Analyzer, Hach Instruments, USA) and nitrate (Tetra, Melle, Germany). Zebrafish were fed three times per day, twice with flake food (TetraMin, Tetra, Germany) and once with Artemia (brine shrimp). Embryos were obtained via natural group spawning (ratio males/females: about 1:1) over marbles, sorted and kept in a petri dish (92 × 16 mm Sarstedt (Nümbrecht, Germany)) at 28 °C in a Peltier-cooled incubator (IPP 260, Memmert, Schwabach, Germany) in Danieau’s solution (1.5 mM HEPES, 17.4 mM NaCl, 0.21 mM KCl, 0.12 mM MgSO4, and 0.18 mM Ca(NO_3_)_2_ and 0.6 μM methylene blue) with a density of 50 embryos per 50 ml. All further experimental work was done using Danieau’s solution as incubation medium. For every experiment, embryos used were collected from the same spawn of eggs.

The zebrafish mutant line “nacre”^50^ with an AB background that lacks melanophores was used throughout the study, except in the case of hepatotoxicity testing where the transgenic zebrafish line *Tg*(*fabp10a:Dsred*) was used that expresses the liver-specific zebrafish fabp10a promoter driving the fluorescent reporter gene DsRed^51^. Embryos and larvae were treated with silica NPs at pre-determined concentrations for a standard period specified for the type of toxicity test (see below). Stock solutions of silica NPs in Milli-Q were sonicated (5 minutes) before adding to Danieau’s medium to make working solutions that were sonicated again (10 minutes) before addition to zebrafish larvae.

### Quantification of silica NP associated with chorion and embryo

Fertilized eggs were exposed to S-20, S-50 and S-80 at a concentration of 200 mg/l from 4hpf to 24hpf. At 24hpf, eggs were washed three times with Danieau’s medium and manually dechorionated. The separated chorions and embryos were washed again and kept at −20 C until further analysis. The amount of silica in the chorions and embryos were measured by ICP-MS as described above. For every experiment, 50 fertilized eggs were used, and the experiment was repeated three times.

### Developmental toxicity

For developmental toxicity testing, fertilized eggs were exposed to silica NPs from 4hpf–120hpf. Developmental toxicity endpoints, including survival, time of hatching, morphological defects were examined every 24 h up to 120hpf (Supplementary Figure S2a). The silica NPs suspensions and control medium were gently refreshed for 40% every 24 h. No mixing prior to removal and replacement was performed in order not to disrupt the hatching process and timing. To score NPs-induced morphological defects, we adopted a semi-quantitative scale^52^ that classifies the abnormality severity into different levels (Supplementary Figure S3a): normal (level 1), mild (level 2), severe (level 3) and death (level 4). 10 fertilized eggs were individually used per condition, and experiments were repeated independently at least three times.

### Hepatotoxicity and cardiotoxicity

For hepatotoxicity and cardiotoxicity testing, *Tg*(*fabp10a:Dsred*) larvae at 96hpf that showed consistent fluorescence in the liver were selected before silica NP exposure. The silica NPs solutions were refreshed for about 40% every 24 h. At 120hpf, larvae were treated briefly with MS-222 (0.16 mg/ml) and the heart rates counted^12^. The heart rates of larvae were normalized against that of control larvae. At 168hpf, larval liver phenotypes were scored blind using fluorescence stereomicroscopy (Leica MZ 10 F). Co-morbidity was also investigated during the experiment (Supplementary Figure S2b). Liver morphology was classified as normal or abnormal, i.e. showing a smaller, reduced, enlarged or rounded livers as indicated in Fig. 3g. 10 larvae were used individually per condition, and experiments were repeated independently three times. Terfenadine 10 μM and paracetamol 10 mM were used as positive controls for testing cardio- and hepatotoxicity, respectively.

### Neurotoxicity

#### Photomotor response

The photomotor response (PMR) is a series of motor behaviors in zebrafish embryos elicited by high-intensity light stimuli^16^. PMR was investigated by automated behavioral tracking according to refs 16,53. Briefly, 4hpf embryos were selected and exposed to silica NPs. At 27hpf, embryos were transferred to a 96-well plate, followed by a dark incubation of 3 hours at 28 °C. To avoid meniscus effects, the wells were completely filled (378 μl volume) and covered with a thin glass plate (0.5 mm thickness). Each well contained 5 fish and three to six wells were used per condition. At 29.5hpf, the 96-well plate was placed in the recording chamber at 28 °C inside the PMR zebrabox (Viewpoint, ZebraLabv3, France) for habituation in the dark. At 30hpf, the PMR-assay was initiated and embryonic motion was recorded by automated behavioral tracking. A group of silica-untreated larvae was also exposed to 100 μM isoprenaline for 3 hours (positive control). The locomotion measured within the 8 different periods that correspond to the pre-stimulus (PRE), latency (L), excitation (E1, E2, E3) and refractory (R1, R2, R3) phases of PMR was calculated. Raw data of total movement per well were used (expressed in motion units) and the data obtained from the different wells per condition averaged. Experiments were repeated independently three times. After recording, a touch response assay was performed.

#### Locomotor response

The locomotor response (LMR) of larvae exposed to light-dark cycles was examined after the developmental toxicity testing (see above, Supplementary Figure S2a). After exposure to silica NPs or control medium (200 μl) from 4hpf to 120hpf or from 96hpf to 120hpf, 10 larvae per condition (i.e. silica concentration, control, …) were individually tracked in wells of a 96-well plate at 27–28 °C using a Zebrabox tracking device (Viewpoint, ZebraLabv3, France) for automated behavioral recording. Recording always occurred between 1 pm–5 pm. A group of untreated larvae was also exposed to 1% ethanol for 30 min (positive control). The light-dark conditions were as described^17^ with some modifications: fish were exposed to a 10 min period of light on and 10 min of light off, and two cycles of 5 min light on and 10 min light of, followed by 5 min of light on. The results from the first 10 min light and dark period were omitted. Experiments were repeated independently three times. First, the raw data of the total movement per minute time interval of 10 larvae per condition (i.e. different silica concentrations, controls) were averaged. These larvae were individually tracked in wells (filled with 200 μl) of a 96-well plate. Next the averages per condition for the combined light (i.e. totally 15 min) and dark conditions (i.e. totally 20 min) of the three experiments were pooled. Finally, the mean of the control values obtained in dark conditions was set to 100% and the results of the other conditions normalized.

After recording, a touch response assay was performed. Only data of larvae scored as “normal” in the semi-quantitative scale after the developmental toxicity were included.

### Statistical analysis

Student t-test or two-way ANOVA analysis with Bonferroni correction using GraphPad Prism 5 were performed to determine the significance of differences between the means. P < 0.05 was considered as statistically significant.

## Additional Information

**How to cite this article**: Pham, D.-H. *et al*. Use of Zebrafish Larvae as a Multi-Endpoint Platform to Characterize the Toxicity Profile of Silica Nanoparticles. *Sci. Rep.* **6**, 37145; doi: 10.1038/srep37145 (2016).

**Publisher’s note:** Springer Nature remains neutral with regard to jurisdictional claims in published maps and institutional affiliations.

## Supplementary Material

Supplementary Information

## Figures and Tables

**Table 1:** Nanomaterials’ toxicity as a function of their physicochemical properties.

Nanomaterials	Zebrafish tests	Toxicity reported	Reference
***Effect of particle size***			
Silica NPs 15 nm and 50 nm	Adult locomotor test	15 nm: reduction of retina and dopaminergic neurons,	46
		15 nm: more severe induction of Parkinson-like behaviors	
Fluorescent core-shell silica NPs 60 nm and 200 nm	Hatching time, mortality, uptake	Both: uptake and translocation in embryos not observed	25
		No induction of changes in hatching time or mortality	
***Effect of particle shape***			
Ni NPs: dendritic vs spherical shape	Survival rate	LC10 value of dendritic Ni NPs < LC10 of free Ni ion < LC10 of spherical Ni NPs	54
SiO_2_: nanowire vs spherical shape	Mortality, morphology	SiO_2_ nanowires: induction of mortality and abnormal phenotypes	55
		SiO_2_ spherical shape: no toxicity	
***Effect of surface properties***			
Au NPs: positive, negative and neutral surface charges	Mortality, particle uptake and retention	Mortality and retention time: positive charged NPs > negative charged NPs > neutral charged NPs (no effect)	56
Ag: coated with citrate or fulvic acid	Mortality	Ag NPs coated with either citrate or fulvic acid: less toxicity	57

**Table 2:** Silica NPs characterization in Danieau’s.

	Day 0		Day 5	
	DLS (PDI) (nm)	Conc (mean ± SD) (mg/l)	DLS (PDI) (nm)	Conc (Mean ± SD) (mg/l)
S-20	28 (0.36)	203 ± 16	30 (0.32)	200 ± 16
S-50	65 (0.20)	186 ± 15	57 (0.16)	213 ± 17
S-80	107 (0.16)	200 ± 16	100 (0.10)	213 ± 17

Silica NPs dilutions in Danieau’s were sonicated (10 min) before addition to zebrafish larvae. The solutions were stored for maximum 5 days. The size of the NPs was characterized by DLS and the nominated concentration (200 mg/l) examined by SAXS.
