# Supplementary material for: Use of Zebrafish Larvae as a Multi-Endpoint Platform to Characterize the Toxicity Profile of Silica Nanoparticles
Source: Sci Rep. 2016 Nov 22;6:37145. doi: 10.1038/srep37145 (PMC5131651; doi:10.1038/srep37145)
Supplement: Supplementary Information [file srep37145-s1.pdf]

USE OF ZEBRAFISH LARVAE AS A MULTI-ENDPOINT PLATFORM TO CHARACTERIZE THE TOXICITY PROFILE OF SILICA NANOPARTICLES

Duc-Hung Pham[1], Bert De Roo[2], Xuan-Bac Nguyen[1], Mattias Vervaele[2], Angela Kecskés[1], Annelii Ny[1] Daniëlle Copmans[1], Hanne Vriens[3], Jean-Pierre Locquet[2], Peter Hoet[3], Peter A.M. de Witte[1*]

1. Laboratory for Molecular Biodiscovery, KU Leuven, Campus Gasthuisberg, Herestraat 49,O&N II, 3000 Leuven, Belgium.

2. Solid State Physics and Magnetism Section, KU Leuven, Department of Physics and Astronomy, Celestijnenlaan 200D, 3001 Heverlee, Belgium.

3. Center for Environment and Health Leuven, KU Leuven, Campus Gasthuisberg, Herestraat 49, O&N I, 3000 Leuven, Belgium.

SUPPLEMENTARY INFORMATION

Table S1: Silica NPs characterization.

CHARACTERIZATIONS	Silica NPs 20nm		Silica NPs 50nm		Silica NPs 80nm	
	manufacturer[a]	this study	manufacturer[a]	this study	manufacturer[a]	this study
Mean TEM diameter (nm)	23.2 ± 2.4	21.8 ± 2.5	50.0 ± 5.3	45.2 ± 4.1	82.6 ± 4.7	81.5 ± 5.2
SAXS diameter (nm)	-	20.3	-	42.9	-	75.2
DLS diameter (nm)	24.9	29.5	62.0	67.6	98.0	107.2
ZP (mV)	-50.8	-25.9	-38.2	-31.5	-43.6	-41.4
ICP-MS (mg/l)	11.6	10.7	10.7	8.6	10.3	9.3

[a]As specified by Nanocomposix in http://nanocomposix.eu/collections/silica

Figure S1

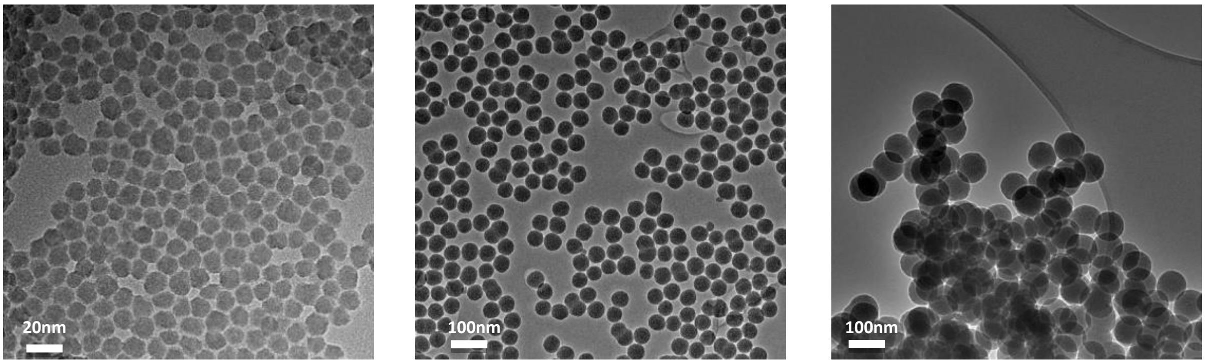

Figure S1: Photomicrographs of 20 nm (left), 50 nm (middle) and 80 nm (right) silica NPs dispersed in Milli-Q water.

Figure S2

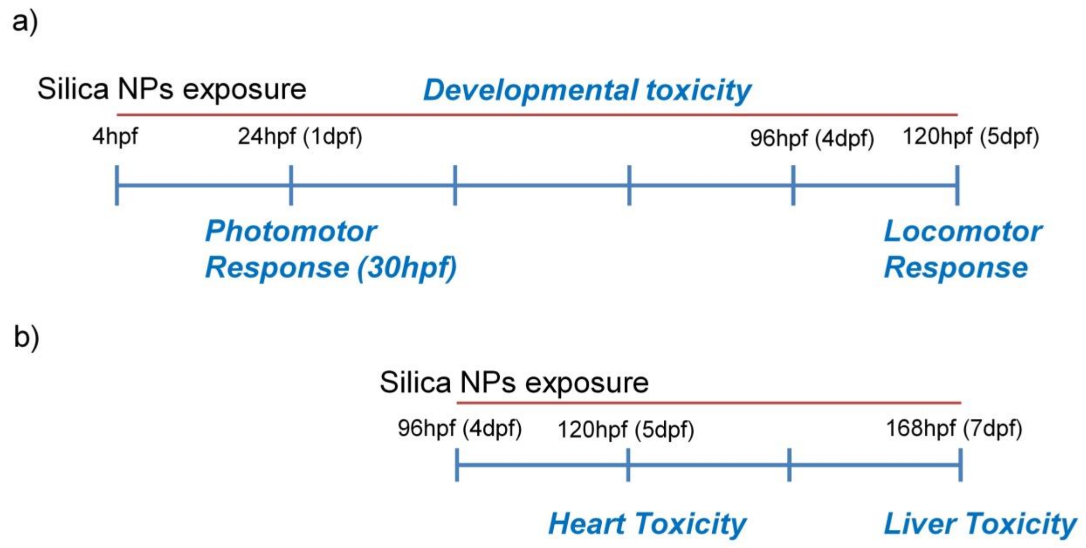

Figure S2: Time scheme of zebrafish exposure to silica NPs and toxicity endpoints. Zebrafish was exposed to silica NPs either from 4hpf – 120hpf for developmental toxicity, photomotor response and locomotor response experiments (a) or from 4dpf – 7dpf for heart and liver toxicity experiments (b). dpf: day(s) post fertilization, hpf: hour(s) post fertilization.

Figure S3

a)

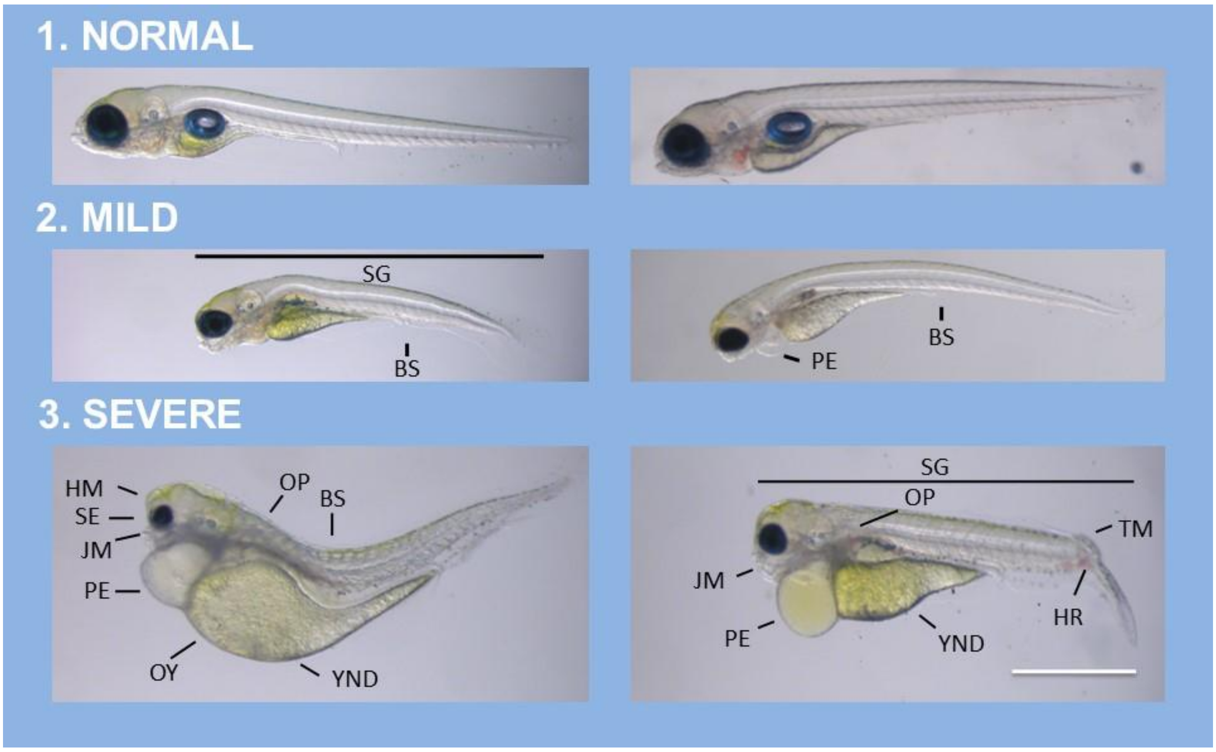

1. NORMAL
2. MILD
SG
BS
BS
PE
3. SEVERE
HM
SE
JM
PE
OY
OP
BS
YND
SG
OP
TM
JM
HR
PE
YND

b)

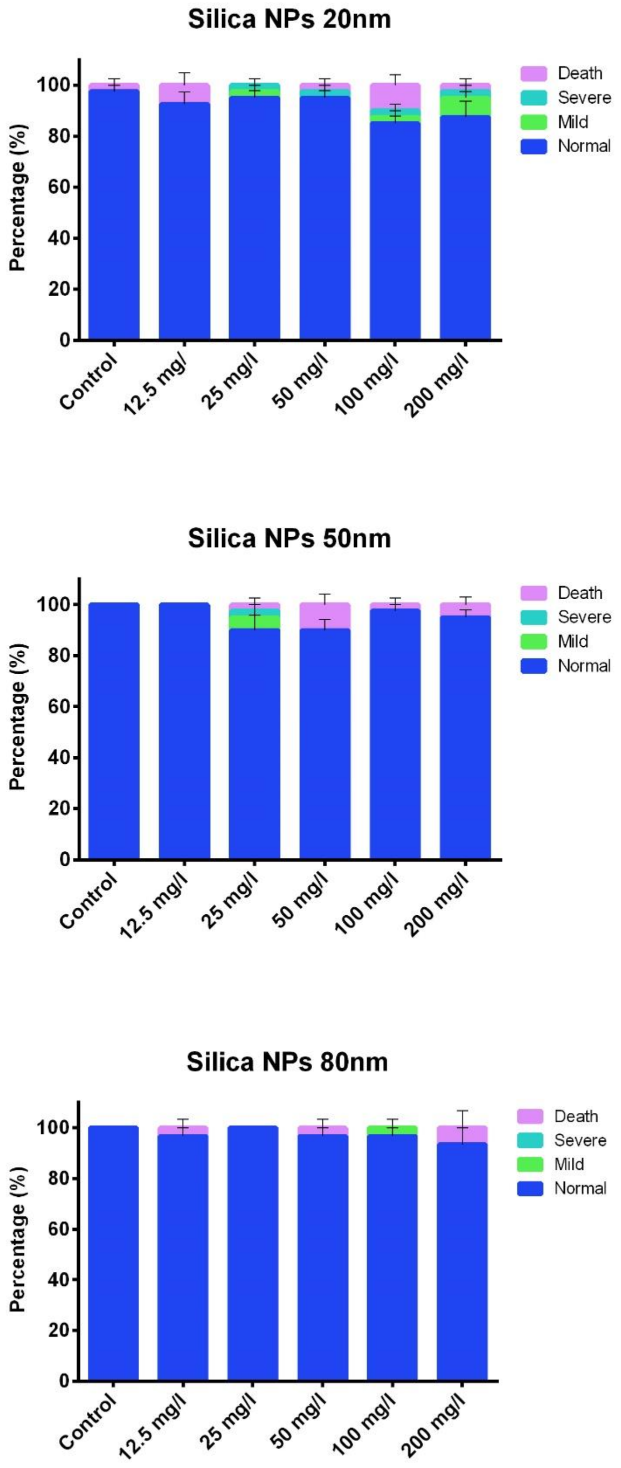
Silica NPs 20nm
Percentage (%)
0
20
40
60
80
100
Control
12.5 mg/
25 mg/l
50 mg/l
100 mg/l
200 mg/l
Death
Severe
Mild
Normal
Silica NPs 50nm
Percentage (%)
0
20
40
60
80
100
Control
12.5 mg/l
25 mg/l
50 mg/l
100 mg/l
200 mg/l
Death
Severe
Mild
Normal
Silica NPs 80nm
Percentage (%)
0
20
40
60
80
100
Control
12.5 mg/l
25 mg/l
50 mg/l
100 mg/l
200 mg/l
Death
Severe
Mild
Normal

Figure S3: Developmental toxicity of silica NPs. Effect of 20 nm, 50 nm and 80 nm silica NPs on the morphology of zebrafish larvae exposed from 4hpf – 120hpf to concentrations ranging from 12.5 mg/ml to 200 mg/ml. a) semi-quantitative scale used to calculate the severity of morphological defects, level 1 -normal: no visual defect detected; level 2-mild: embryos/larvae have less than 4 mild defects, level 3 -severe: embryos/larvae have at least one severe defect or more than 3 mild malformations; and level 4-death. PE: pericardiac edema, HM: head malformation, SE: small eyes, OP: opaque tissue, OY: opaque yolk, YND: yolk not depleted, BS: bent spine, JM: jaw malformation, SG: slow growth, TM: tail malformation, HR: hemorrhage. Scale bar = 1 mm; (b) Percentage of larval fish that showed a normal, mild or severe developmental phenotype or were dead at 120 hpf. 10 eggs were used individually per condition. Data represent the means and standard error of the mean for three independent biologic replicates. ($p^*<0.05$, two-way ANOVA, Bonferroni correction).

Figure S4

a)

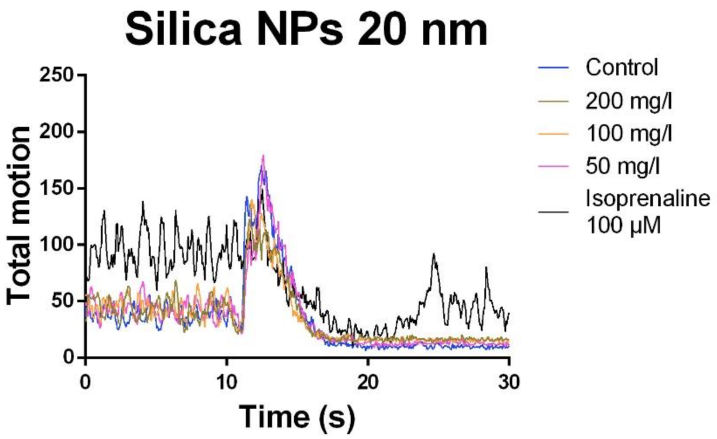

b)

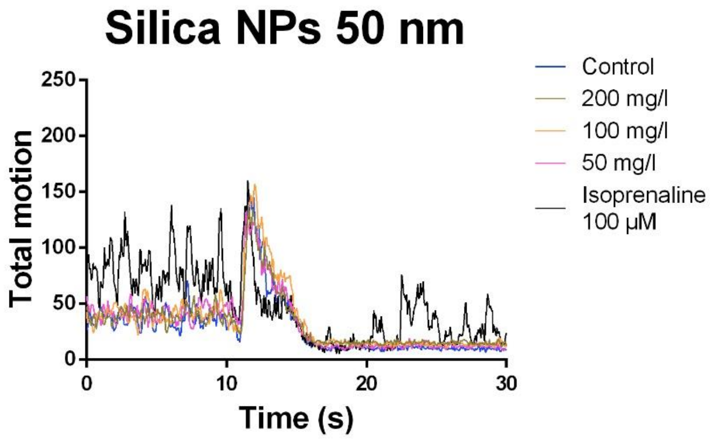

c)

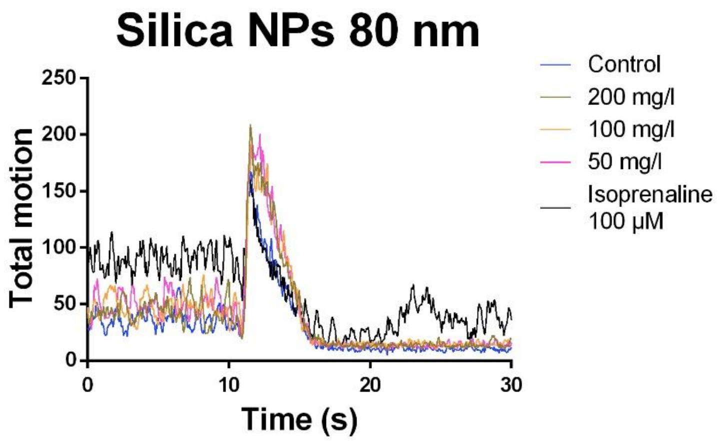

Figure S4: Photomotor response of 30hpf zebrafish larvae exposed from 4hpf – 30hpf to 20 nm (a), 50 nm (b) and 80 nm (c) silica NP concentrations ranging from 50 mg/ml to 200 mg/ml. The data show the real time locomotion recorded during the duration of the experiment expressed as motion units (y axis) versus time in second (x axis). For reasons of clarity, data in (a), (b) and (c) are presented as mean of data only.

Figure S5

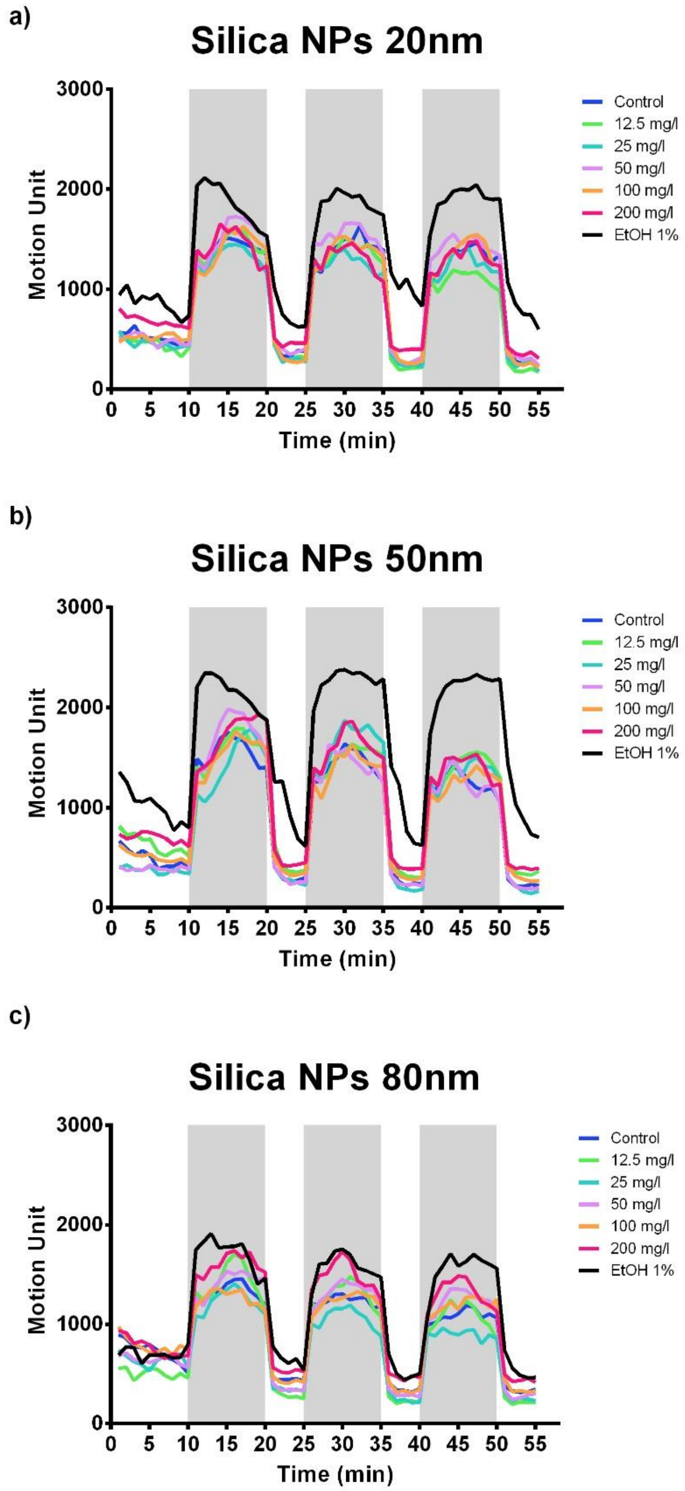
a)
Silica NPs 20nm
b)
Silica NPs 50nm
c)
Silica NPs 80nm
Motion Unit
0
1000
2000
3000
Time (min)
0 5 10 15 20 25 30 35 40 45 50 55
Control
12.5 mg/l
25 mg/l
50 mg/l
100 mg/l
200 mg/l
EtOH 1%

Figure S5: Locomotor response of 120hpf zebrafish larvae exposed from 4hpf – 120hpf to 20 nm (a), 50 nm (b) and 80 nm (c) silica NP concentrations ranging from 12.5 mg/ml to 200 mg/ml. 1 % ethanol was used as positive control. The data show the real time locomotion recorded during the time of the experiment expressed as motion unit (y axis) versus time by minute (x axis). For reasons of clarity, data in (a), (b) and (c) are presented as mean of data only. The white background indicate light periods and dark background indicate dark periods.

Figure S6

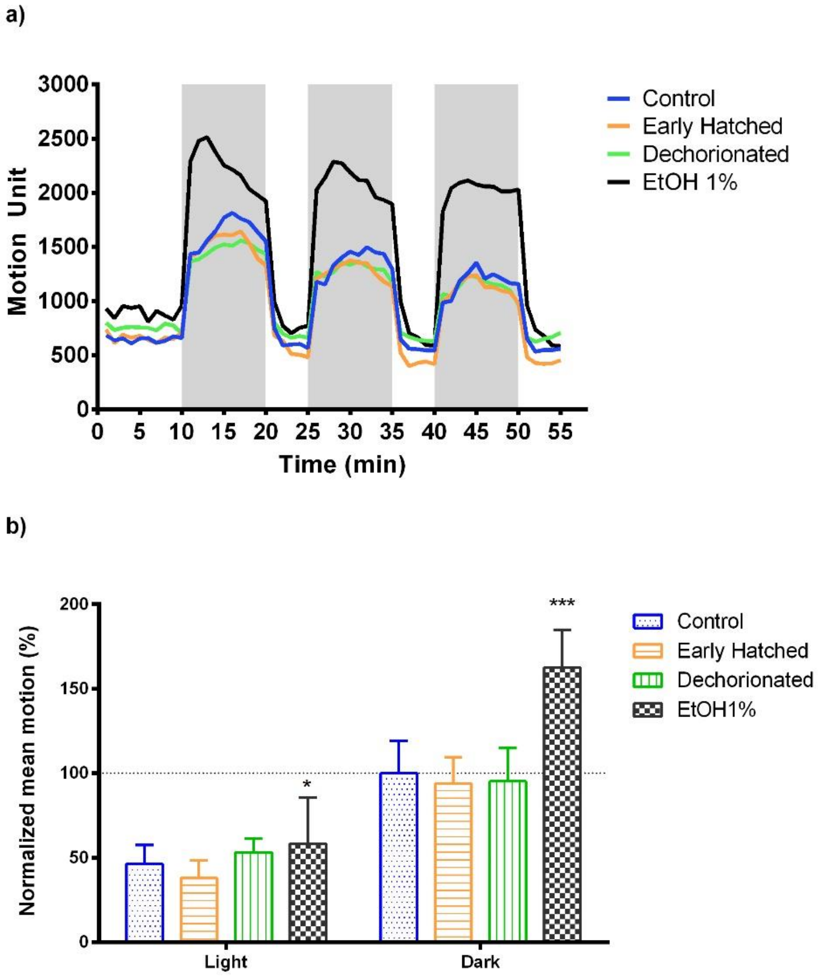

Figure S6: Early hatching or dechorionation does not alter locomotor response of 120hpf zebrafish larvae. (a) the data show the real time locomotion recorded during the time of the experiment expressed as motion unit (y axis) versus time by minute (x axis). 1 % ethanol was used as positive control. For reasons of clarity data are presented as mean of data only. The white background indicate light periods and dark background indicate dark periods. (b) The locomotion (distance/minute) during the three 5 min light periods and the two 10 min dark periods were pooled. 10 larvae were individually used per condition, the results per condition averaged and normalized against that of control larvae. 1 % ethanol was used as a positive control. Data represent the means and standard deviations for at least three independent biologic replicates. (two-way ANOVA, Bonferroni correction)

Figure S7

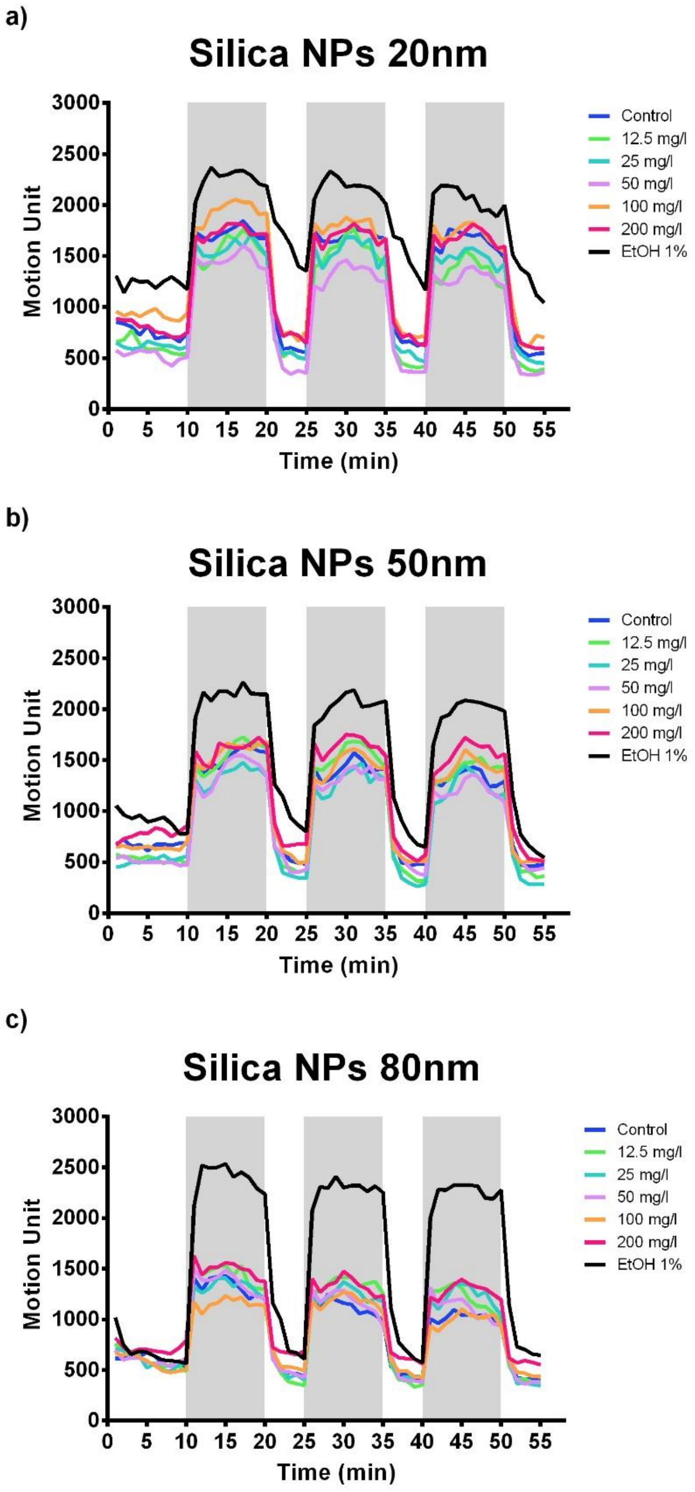
a)
Silica NPs 20nm
Motion Unit
Time (min)
Control
12.5 mg/l
25 mg/l
50 mg/l
100 mg/l
200 mg/l
EtOH 1%
b)
Silica NPs 50nm
Motion Unit
Time (min)
Control
12.5 mg/l
25 mg/l
50 mg/l
100 mg/l
200 mg/l
EtOH 1%
c)
Silica NPs 80nm
Motion Unit
Time (min)
Control
12.5 mg/l
25 mg/l
50 mg/l
100 mg/l
200 mg/l
EtOH 1%

Figure S7: : Locomotor response of 120hpf zebrafish larvae exposed from 96hpf – 120hpf to 20 nm (a), 50 nm (b) and 80 nm (c) silica NP concentrations ranging from 12.5 mg/ml to 200 mg/ml. 1 % ethanol was used as positive control. The data show the real time locomotion recorded during the time of the experiment expressed as motion unit (y axis) versus time by minute (x axis). For reasons of clarity, data in (a), (b) and (c) are presented as mean of data only. The white background indicate light periods and dark background indicate dark periods.
